# Application of Nanomaterials to Enhance Polymerase Chain Reaction

**DOI:** 10.3390/molecules27248854

**Published:** 2022-12-13

**Authors:** Zhu Yang, Bei Shen, Lihuan Yue, Yuqing Miao, Yihong Hu, Ruizhuo Ouyang

**Affiliations:** 1 Institute of Bismuth and Rhenium Science, School Materials and Chemistry, University of Shanghai for Science and Technology, Shanghai 200093, China; 2 Institut Pasteur of Shanghai, Chinese Academy of Sciences, University of Chinese Academy of Sciences, Shanghai 200031, China; 3 CAS Key Laboratory of Molecular Virology & Immunology, Institutional Center for Shared Technologies and Facilities, Pathogen Discovery and Big Data Platform, Institute Pasteur of Shanghai, Chinese Academy of Sciences, Shanghai 200031, China; 4 School of Life Sciences and Biotechnology, Shanghai Jiao Tong University, Shanghai 200240, China

**Keywords:** NanoPCR, nanomaterials, specificity, efficiency, mechanisms

## Abstract

Polymerase Chain Reaction (PCR) is one of the most common technologies used to produce millions of copies of targeted nucleic acid in vitro and has become an indispensable technique in molecular biology. However, it suffers from low efficiency and specificity problems, false positive results, and so on. Although many conditions can be optimized to increase PCR yield, such as the magnesium ion concentration, the DNA polymerases, the number of cycles, and so on, they are not all-purpose and the optimization can be case dependent. Nano-sized materials offer a possible solution to improve both the quality and productivity of PCR. In the last two decades, nanoparticles (NPs) have attracted significant attention and gradually penetrated the field of life sciences because of their unique chemical and physical properties, such as their large surface area and small size effect, which have greatly promoted developments in life science and technology. Additionally, PCR technology assisted by NPs (NanoPCR) such as gold NPs (Au NPs), quantum dots (QDs), and carbon nanotubes (CNTs), etc., have been developed to significantly improve the specificity, efficiency, and sensitivity of PCR and to accelerate the PCR reaction process. This review discusses the roles of different types of NPs used to enhance PCR and summarizes their possible mechanisms.

## 1. Introduction

Polymerase chain reaction (PCR) technology, first proposed by Mullis in the United States in 1983 and invented in 1985, is a molecular biology technique used to amplify specific DNA fragments and is regarded as a unique DNA replication in vitro. The most prominent feature of PCR is that it can significantly increase the trace amount of DNA. So far, it has been widely used in many different fields, such as medical diagnosis [1], food safety [2], archaeological research [3], basic bioresearch, etc. However, the development of PCR is subject to certain limits owing to low specificity, efficiency, and sensitivity. Although some important parameters in PCR have been optimized to improve its specificity and efficiency, including polymerase concentration, annealing temperature, cycle number, template type, primer design, and magnesium ion concentration, the effect is still not satisfactory [4]. Nanotechnology, therefore, has been applied to improve the performance of PCR. At present, many nanomaterials have been successfully used to enhance the specificity and efficiency of PCR, such as Au NPs [5], QDs [6], CNTs [7], graphene oxide (GO) and reduced GO (rGO) [8], partial metal oxidation materials [9,10] (e.g., titanium dioxide, zinc oxide) and other composite materials like macromolecule polymer doped with Au NPs [11], amino-modified semiconductor magnetic NPs [12], and so on.

Nanomaterials are composed of particles with at least one external dimension less than 100 nm, and they have been widely used in electronics, aerospace, military, chemical, biomedical, and healthcare products [13]. In PCR, nanomaterials are added to the reaction system that mainly contains primers, enzymes, and templates, due to the characteristics of nanomaterials and their role in PCR. Here, numerous NPs were divided into three categories according to the effects of nanoparticles in PCR. The first type of nanomaterial, such as Au NPs, GO, carbon nanopowder (CNP), etc., has good thermal conductivity, which could speed up the process and shorten the reaction time of the original reaction procedure, therefore enhancing the efficiency of PCR. The second one, which includes CNTs, magnetic NPs, polymer-modified silica, QDs, etc., may interact with the surface of nanomaterials via van der Waals forces among the reaction system components, or provide many binding sites to fix polymerases, so that the added template and material form a competitive relationship, and thus enhance the specificity of PCR. The third type includes polymer-modified gold, GO, CNTs, ZnO with amino groups, etc., which have a positive surface charge and attract negatively charged nucleotide chains (templates and primers) containing phosphate groups, thereby enhancing the specificity of PCR. Of course, the enhancement of each type of NP described here in PCR is not just a cause, it is the main enhancement mechanism. Some NPs create catalytic activity or similar to ssDNA-binding proteins (SSB), which have the characteristics of selective adsorption of ssDNA. This is often caused by a variety of factors.

However, the mechanisms of nanoPCR are still unclear because of the complexity of the PCR system and characteristics of NPs. The possible mechanisms are described as follows: (1) surface interactions between nanomaterials and PCR components; (2) thermal conversion rate of nanomaterials; (3) electrostatic interaction; (4) being analogous to ssDNA binding proteins (SSB); and (5) catalytic activity. Undoubtedly, these mechanisms cannot explain clearly the impact of all nanomaterials on PCR. Therefore, more undiscovered mechanisms need to be thoroughly explored. Herein, this review aims to discuss the applications of different nanomaterials in PCR and summarize the possible mechanisms between various nanomaterials and the PCR components.

## 2. Utilizing Different Nanomaterials to Enhance PCR Effects

### 2.1. Metal Nanomaterials

#### 2.1.1. Au NPs

**Figure 1:**
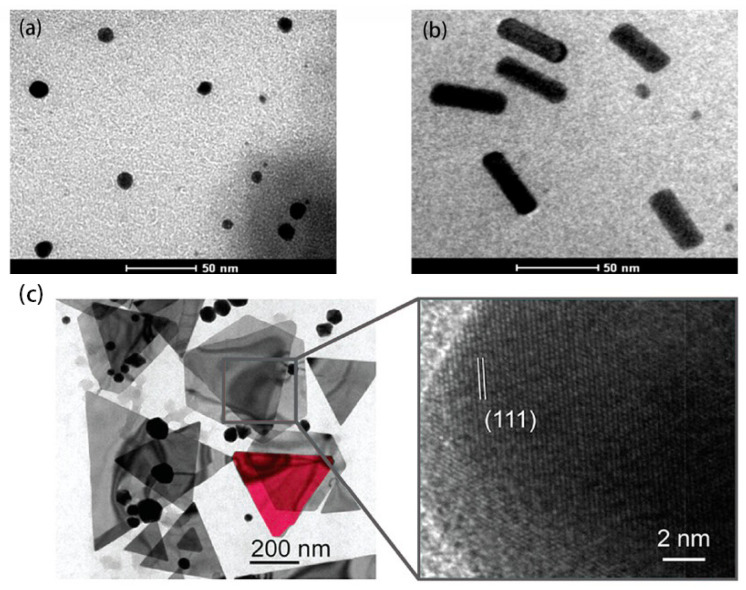
TEM images of (**a**) spherical, (**b**) rod (Reproduced with permission from Ref. [15], Copyright 2022, Elsevier), and (**c**) **triangular** Au NPs. (Reproduced with permission from Ref. [16], Copyright 2020, American Chemical Society).

Au NPs are the most well-studied nanoparticles, and their interesting chemical and photophysical properties make them an integral part of nanoscience and ideal for biological and other commercial laboratories, which contain non-toxic, good biocompatibility, and unique chemical and optoelectronic properties. For example, Au NPs are characterized by adjustable size and physical size, catalytic activity, high surface volume ratio, high stability, easy synthesis and surface modification, and strong light absorption and scattering properties [5,14]. Figure 1 shows transmission electron microscopy (TEM) images of Au NPs in different morphologies [15,16].

Li et al. for the first time reported the optimization of Au NPs on PCR. The Au NPs with a particle size of about 13 nm were proved to dramatically improve the efficiency of PCR. Compared with the reagent without Au NPs, the amplification yield of PCR reagent with Au NPs increased at least 10^4^ to 10^6^-fold with shortened PCR time in testing different sizes of the DNA fragments [17]. Subsequently, Pan et al. studied the interaction mechanism of Au NPs and DNA polymerase in the PCR system. It was found that Au NPs could optimize the PCR amplification strategy and inhibit the nonspecific amplification of PCR. The amplification limit of detection (LOD) increased approximately seven-fold [18]. In 2008, Binh et al. found that the effect of Au NPs was not to increase PCR specificity but to favor smaller products over more oversized products, regardless of specificity. Such an effect could be duplicated simply by reducing polymerase concentration but be reversed by increasing polymerase concentration or adding BSA as a competitive displacer [19]. The study of the interaction between Au NPs and DNA polymerase indicated that the addition of DNA polymerase could eliminate the inhibitory effects of the excess Au NPs, and in the reverse, Au NPs could eliminate the inhibitory effects of the excess DNA polymerase [20]. Moreover, Au NPs have been proven to be able to simultaneously enhance both PCR efficiency and specificity by improving the thermal conductivity of the PCR solution [21]. Afterwards, Lou et al. generally summarized the three effects of Au NPs on PCR: (1) Au NPs adsorbed polymerase; (2) Au NPs decreased the melting temperatures (Tm) of both complementary and mismatched primers and increased the Tm difference between them; and (3) Au NPs facilitated the dissociation of the PCR products in the denaturing step [22]. Mandal et al. proposed that the enhancement of PCR yield by Au NPs with a particle size of 11 nm could be attributed to the greater affinity and thermodynamic stability of Au NPs for Taq DNA polymerase compared to the primer or DNA template [23].

#### 2.1.2. Ag NPs

**Figure 2:**
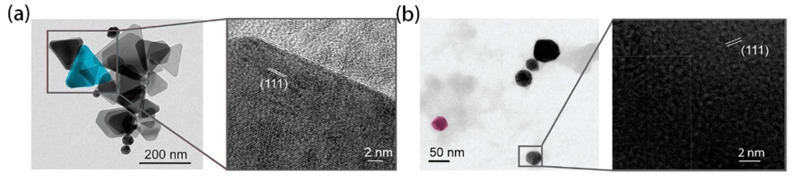
(**a**) Triangular and (**b**) spherical Ag NPs with lattice orientation zoomed image. (Reproduced with permission from Ref. [16], Copyright 2020, American Chemical Society).

At present, the use of metallic silver, silver nitrate, and silver sulfadiazine to treat burns, wounds, and bacterial infections has significantly declined because of the emergence of antibiotics. With the tremendous impetus of nanotechnology gains, nano-sized silver (Ag NPs) shows dramatically diverse chemical, physical, and optical properties and has high optical tunability, large absorption cross sections, and scattering properties [16]. Figure 2a,b are the TEM images of triangular NPs and spherical Ag NPs, respectively.

Wang et al. found that the presence of Ag NPs could significantly retain the specificity of long PCR products after three rounds of repeated amplification [24]. Liu et al. studied the effect of Ag NPs on DNA synthesis in PCR where Ag NPs over a certain size and concentration significantly inhibited PCR amplification [25]. Recently, Kadu et al. reported the effect of the shape of Ag NPs on photothermal properties and PCR efficiency. Triangular Ag NPs were able to increase PCR efficiency [16].

### 2.2. Carbon-Based Nanomaterials

#### 2.2.1. CNTs

**Figure 3:**
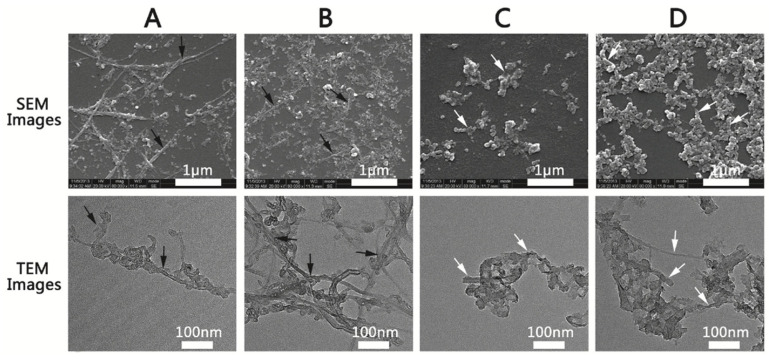
The SEM and TEM images of (**A**) unmodified SWCNTs, (**B**) modified SWCNTs, (**C**) unmodified MWCNTs, and (**D**) modified MWCNTs. The black and white arrows refer to SWCNTs and MWCNTs, respectively. (Reproduced with permission from Ref. [27], Copyright 2018, Japanese Society for Dental Materials and Devices).

CNTs are greatly advantageous because of high electron transport without electronic scattering and electronic conductivity; thus, they provide high-performance sensing transistors. More specifically, CNTs possess a high aspect ratio (the ratio of lateral size to thickness), large specific surface area (SWCNT > 1600 m^2^/g, MWCNT > 430 m^2^/g), as well as good mechanical and electrical (~5000 s/cm) properties [26,27]. The SEM and TEM images of SWCNTs and MWCNTs are shown in Figure 3.

Cui et al. first reported the positive effect of CNTs on PCR amplification. The addition of single-walled CNTs (SWCNTs) into the reaction liquid increased the amount of PCR product at a SWCNT concentration of 3 μg μL^−1^, but reversed at SWCNT concentrations higher than 3 μg μL^−1^ [7]. The beneficial effect of both SWCNTs and multiwalled CNTs (MWCNTs) was also reported to enhance the specificity and total efficiency of long PCR (14 kb). The hydroxylic and carboxylic CNTs had similar enhancing effects as well. Moreover, various functional groups and polymer-modified CNTs also played an even more substantial role in enhancing PCR amplification [28]. The PEI-modified MWCNTs with different surface charge polarities as a novel class of enhancers were successfully used to improve the specificity and efficiency of PCR. Positively charged PEI-modified MWCNTs (CNT/PEI) significantly enhanced the specificity and efficiency of PCR at an optimum concentration as low as 0.39 mg L^−1^, whereas neutral CNT/PEI modified with acetic anhydride (CNT/PEI.Ac) had no such effect. Although the negatively charged CNT/PEI modified with succinic anhydride (CNT/PEI.SAH) could enhance the PCR, the optimum concentration required (630 mg L^−1^) was over three orders of magnitude higher than that of the positively charged CNT/PEI [29]. On the other hand, the amine functionalized MWCNT (NH_2_-MWCNT) dispersion enhanced total PCR efficiency up to 70% after being sonicated, centrifuged, and filtered, while NH_2_-MWCNTs inhibited the reaction significantly at similar concentrations without being filtered [30]. The study of three kinds of CNTs containing pristine, amine-functionalized, and carboxyl-functionalized SCNTs showed that both the pristine and the amine-functionalized SCNTs could enhance the final amplification yields of the samples. However, the carboxylated SCNTs displayed an inhibitory action in all samples [31].

#### 2.2.2. CNP

**Figure 4:**
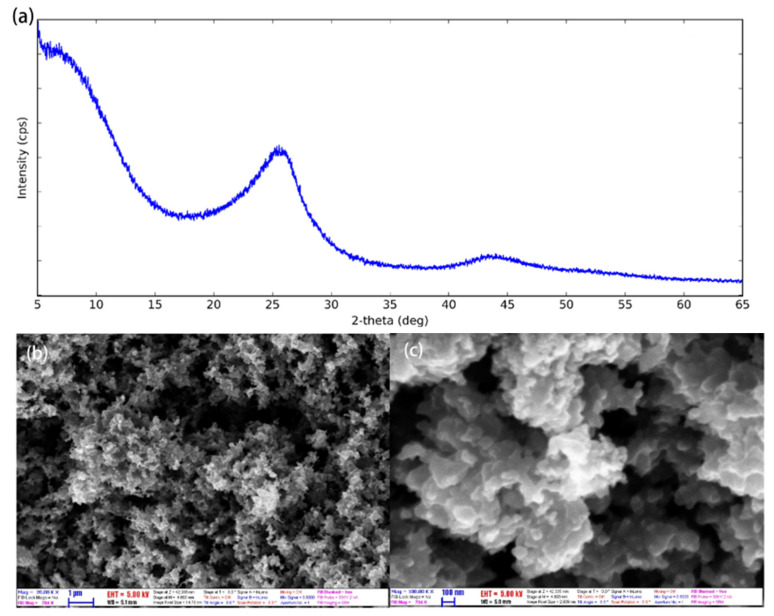
(**a**) XRD pattern of CNP at the following XRD conditions: X-ray: 40 kV, 30 mA. Scan speed: 3.0 degree/min. (**b**,**c**) SEM images of CNP with magnification ×20,000 and ×100,000. (Reproduced with permission from Ref. [32], Copyright 2021, MDPI).

CNP has high specific surface area, strong adsorption, and high electrochemical capacity. As seen in Figure 4a, CNP has two broad peaks at 2θ of 25° and 43.8°, respectively. The diffraction peaks correspond to the planes (002) and (101) of graphite, indicating either a high degree of graphitization or a high degree of crystallinity, which can increase the thermal conductivity of CNP nanofluids due to the amorphous particles scatter phonon. This is probably the main reason why it enhances PCR. Figure 4b,c show the SEM photographs of the morphology of CNP. Clearly, the CNP is irregular, and the particles tend to aggregate with the diameter of CNP around 60 nm [32].

Over ten years ago, carbon nanopowder was proven for the first time to have a beneficial effect on enhancing the efficiency of PCR amplification in a repeated PCR and a long PCR system. For the repeated PCR, the addition of a certain amount of CNP could obtain the target products even in sixth-round amplification with high specificity dependent on the concentration of CNP. The CNP significantly improved the amplification efficiency for long PCR reactions [33].

#### 2.2.3. Graphene

Graphene, known as a 2D crystal of sp^2^-hybridized carbon atoms arranged in six-membered rings, has an extensive theoretical specific area, unparalleled thermal and electricity conductivity, and fascinating electronic properties such as an ambipolar electric field effect along with ballistic conduction of charge carriers [34]. However, during the preparation of GO, the oxygen-containing functional groups are usually introduced on the surface of graphene, and these heteroatoms will combine with adjacent carbon atoms through covalent bonds or weak van der Waals forces, resulting in a sharp decrease in thermal conductivity due to high-density defects caused to graphene [35]. Therefore, the thermal conductivity better enhances the properties of rGO than GO in PCR.

In the study of graphene-enhanced PCR, Jia et al. found that the specificity of the PCR amplification could be improved by adding GO at concentrations from 12 mg∙mL^−1^ to 60 mg∙mL^−1^. However, GO did not affect the PCR when the GO concentration was lower than 12 mg∙mL^−1^, while it exhibited an inhibitory effect at concentrations higher than 70 mg∙mL^−1^. This study first demonstrated that rGO could significantly improve PCR specificity. It was then concluded that rGO was superior to GO in enhancing specificity [36]. Wang et al. further demonstrated that 1 μg∙mL^−1^ of GO effectively enhanced the specificity of the error-prone multi-round PCR [8]. In addition to conventional graphene, Abdul et al. explored the effect of graphene nanoflakes (GNFs) on PCR and found that 0.01% (*w*/*w*) GNFs provided an unambiguous 10-fold enhancement in the PCR yield. In addition, the thickness of the GNFs had a significant impact on the yield of PCR products. The 8 nm-thick GNFs increased the yield higher than other sizes [37]. Recently, Zhong et al. discussed the effects of GO through surface modification on PCR. The zwitterionic polymer-modified GO was found to be superior to other GO derivatives, with different charges enhancing the specificity of PCR [38].

### 2.3. Oxide Nanomaterials

#### 2.3.1. TiO_2_

TiO_2_ has been known as one of the cheapest and most widely-available types of NPs utilized for thermal conductivity enhancement [39]. Murshed et al. [40] demonstrated that TiO_2_ NPs have wonderful physical and chemical stability. It has been found that their particle size, shape, and volume fraction are the most critical factors that contribute to enhanced thermal conductivity.

Both size and concentration of TiO_2_ NPs affects PCR. It was found that TiO_2_ NPs inhibited DNA synthesis in vitro more severely than the TiO_2_ particles in microscale at the equivalent concentration and the inhibition effect of TiO_2_ NPs was concentration-dependent in the dark [9]. About a decade ago, Rak et al. observed that TiO_2_ NPs with ∼25 nm diameter caused significant enhancement of PCR efficiency for various types of templates. The optimal concentration was determined to be 0.4 nM, resulting in up to a seven-fold increase in the amount of PCR product. As much as a 50% reduction in overall reaction time was also achieved by utilizing TiO_2_ NPs without compromising the PCR yield [41]. Upon the addition of TiO_2_ NPs with a particle size of 7 nm to the ordinary PCR, RT-qPCR, and RT-PCR (reverse transcription PCR), the effects of TiO_2_ NPs were investigated. The results indicated that 0.2 nM TiO_2_ NPs could achieve target amplification at a very low template concentration in an ordinary PCR system. Furthermore, relative to the larger TiO_2_ particles (25 nm) used in a previous study, the smaller TiO_2_ particles (7 nm) used in this study increased the yield of PCR by three-fold or more [42].

#### 2.3.2. ZnO

**Figure 5:**
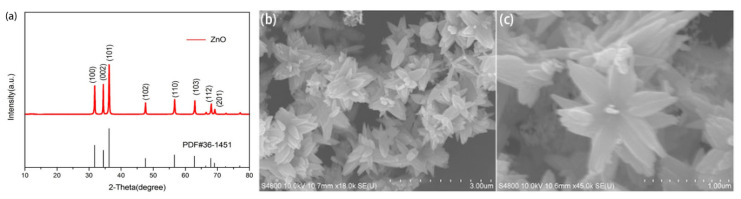
(**a**) XRD patterns of ZnO nanoflowers. SEM images of ZnO: (**b**) low magnification with a diameter of 3.00 μm, (**c**) high magnification with a diameter of 1.00 μm. (Reproduced with permission from Ref. [44], Copyright 2020, MDPI).

ZnO has been widely studied because it is non-toxic and easy to synthesize. Up to now, powdery ZnO in various morphologies, including nanowires, nanoflowers, and spherical and hierarchical structures have been successfully prepared and used to study their photocatalytic properties [43]. ZnO nanoflowers and their composites have been effectively used for PCR [44]. The XRD patterns and SEM images of ZnO nanoflowers are shown in Figure 5. The diffraction peaks are exactly the same as the standard card in the ZnO powder diffraction file (PDF) #36-1451. This clearly shows that the synthesized ZnO nanoflowers are of high purity. The SEM images show that the synthesized ZnO nanoflowers are self-assembling and clearly depict the nanopetal-like structure that emerges from the center of the flower. The synthesized ZnO nanoflowers are clear, uncrowded, and well dispersed, with an average diameter of about 1–2 μm [44].

The tetrapod-shaped SiO_2_-coated ZnO nanostructure with amino groups on the surface was first discovered to have a positive effect on PCR and could increase the yield of PCR amplification [10]. The incorporation of the ZnO nanoflowers in PCR led to a drastic improvement in the efficiency and yield of the ZnO nanoflower-assisted PCR, and reduced the time to perform the PCR assay [44].

#### 2.3.3. Fe_3_O_4_

Magnetic NPs like Fe_3_O_4_ are characteristic of good magnetization and super-para-magnetism. Compared with other nanomaterials, the surface of magnetic NPs is more able to be functionalized.

**Figure 6:**
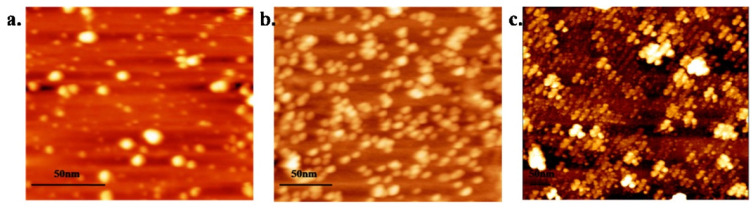
AFM images (**a**) Ag, (**b**) Au and (**c**) magnetite NPs, respectively. (Reproduced with permission. from Ref. [45], Copyright 2016, Elsevier).

For instance, Fe_3_O_4_ nanomaterials have been found to be able to improve the sensitivity of PCR with a detection limit reaching 4.26 mol∙L^−1^. Kambli et al. compared the PCR efficiency enhanced by three transition metal NPs in the form of stable colloidal suspensions at varying concentrations. The AFM images of three nanoparticles are shown in Figure 6. Compared to the citrate stabilized Ag NPs (25 nm, 45%) and Au NPs (15.19 nm, 134%), the highest amplification efficiency of 190% was received using the ammonium salt of oleic acid-coated Fe_3_O_4_ NPs with an average size of 33 nm at a concentration of 7.2 × 10^−3^ nM in a conventional PCR system [45].

**Figure 7:**
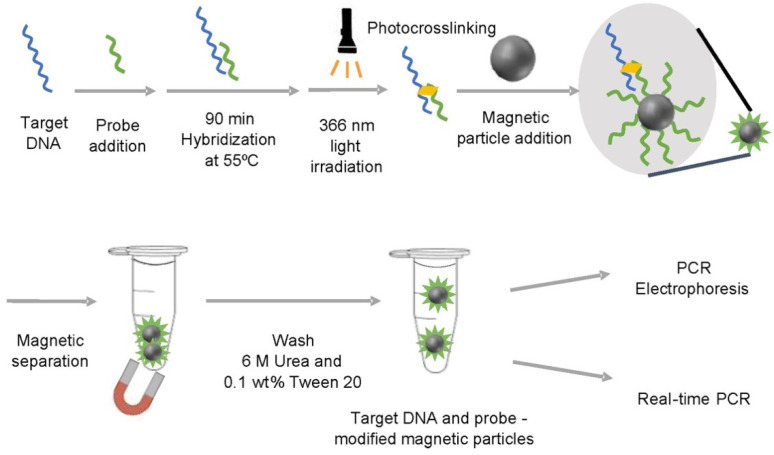
Recovery of target nucleic acids using photo-cross-linkable artificial nucleic acid probes. (Reproduced with permission from Ref. [47], Copyright 2022, American Chemical Society).

Ozalp et al. synthesized magnetic core-silica shell NPs for easy one-step fixation of Taq polymerase directly from crude extract formulations. The magnetic properties of the pellets facilitate rapid purification to eliminate inhibitory elements present in the crude extract during Taq polymerase isolation. They found that at room temperature, after one month, the common Taq enzyme lost about 50% of the cationic activity of the amplification product, while the Taq-silica hybrid retained its original activity for about five months. Additionally, by recovering the Taq polymerase immobilized on the magnetic silica nanoparticles, repeated PCR was performed, and it was found that the immobilized enzymes still retained their original activity after four cycles, although their activity decreased to 45% after seven cycles [46]. Recently, Yajima et al. successfully developed photo-cross-linkable probe-modified magnetic particles (PPMPs) for sequence-specific recovery of target nucleic acids using optical cross-linkable artificial nucleic acid probes and magnetic particles. PPMPs were prepared by adding biotin to the end of the photo-cross-linkable probe following affinity binding with streptavidin-coated magnetic beads. Nucleic acid probes modified with photo-cross-linked artificial nucleic acids can hybridize to the nucleic acid of interest in a sequence-specific manner and then firmly capture the nucleic acid of interest by covalent bonding mediated by UV irradiation. Then, the target nucleic acid is detected by trapping the target-bound probe on the surface of the magnetic particles and subjecting these collected magnetic particles to PCR so as to improve the sensitivity of the PCR detection (Figure 7) [47].

#### 2.3.4. MgO

MgO nanomaterials have unique properties such as being highly stable with good dispensability and less toxic effects. For example, Narang et al. introduced MgO NPs to a PCR system and caused significant improvement in PCR efficiency [48].

#### 2.3.5. SiO_2_

**Figure 8:**
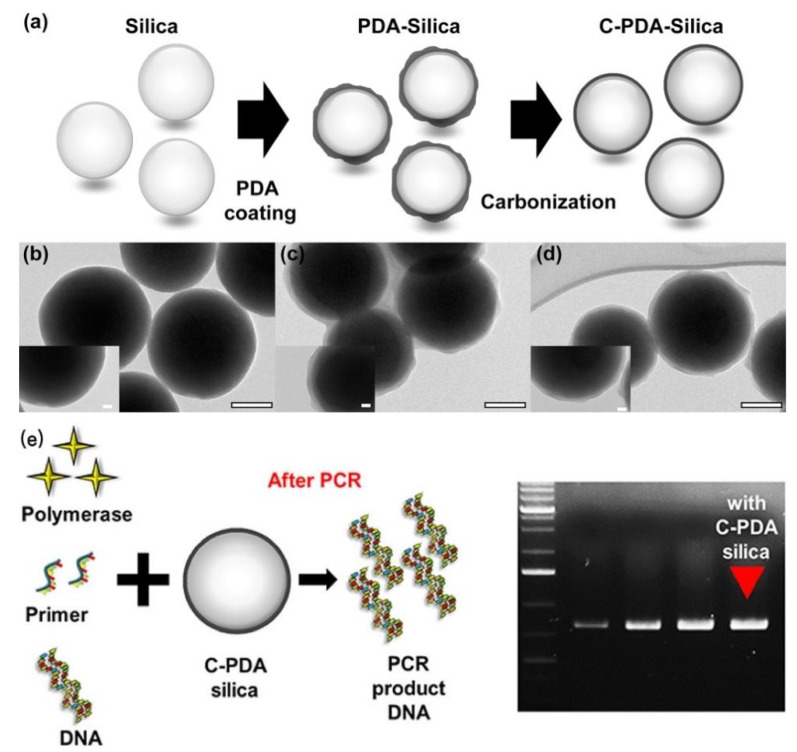
(**a**) Schematic illustration of the synthesis process of silica, PDA silica, and C-PDA silica and (**b**) their corresponding TEM images. All scale bars in (**b**–**d**) are 100 nm for the main panels and 20 nm for the insets, respectively. (**e**) C-PDA silica was employed to increase the PCR efficiency. (Reproduced with permission from Ref. [49], Copyright 2015, American Chemical Society).

SiO_2_ nanomaterials with well-defined morphology and porosity were first prepared and characterized by Stober. Carbonized polydopamine silica (C-PDA silica) were synthesized and employed to increase PCR efficiency (Figure 8). As compared with the effects of SiO_2_ NPs and PDA silica on PCR, C-PDA silica exhibited about 1.5 and 1.2 times higher efficiency. As a result, C-PDA silica can not only reduce the PCR cycle but also increase the final quantity of the PCR product [49].

### 2.4. Fluorescent Nanomaterials

#### 2.4.1. QDs

QDs, as a new kind of fluorescent material, possess many excellent characteristics such as size-tunable emission, wide absorbance bands, narrow symmetric emission bands, high photostability, etc.

In 2009, Wang et al. [6] first found that CdTe QDs could increase the specificity of the PCR at different annealing temperatures with DNA templates of different lengths. Also, CdTe QDs were reported to accelerate PCR speed [50]. Then a Pfu polymerase based multi-round PCR technique was developed through being assisted by CdTe QDs, and the specificity could be retained even in ninth-round amplification [51]. The stacking of the primers on graphene QDs(GQDs) could improve the sensitivity and specificity of PCR by improving the efficiency of base-pairing between the primer and the template. By increasing polymerase activity, GQDs could improve the yield of PCR, where GQDs are tuned through chelating magnesium ions with their peripheral carboxylic groups [52].

#### 2.4.2. Up-Conversion Nanomaterials

**Figure 9:**
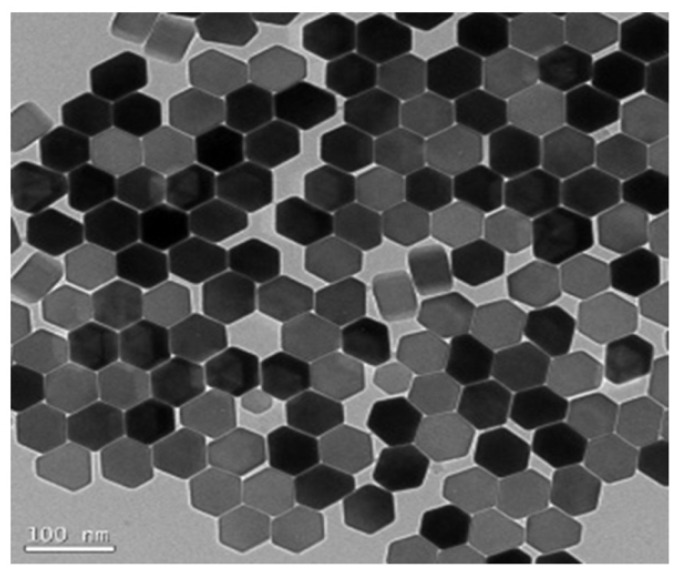
TEM image of 70 nm bare UCNPs. (Reproduced with permission from Ref. [53], Copyright 2013, Public Library of Science).

Photon up-conversion is the phenomenon where high-energy photons are emitted upon the excitation of low-energy photons (Figure 9). Nucleic acid detection based on up-conversion NPs (UCNPs) displays a high signal-to-noise ratio and no photobleaching and has been widely applied. For example, Wang et al. [53] demonstrated that the addition of UCNPs to the reaction mixtures at appropriate concentrations could improve PCR specificity.

### 2.5. Others

#### 2.5.1. Hybrid Nanocomposites

In the past decade, the application of composite nanomaterials in PCR has emerged to optimize the disadvantages of nanoparticles such as easy aggregation, poor adsorption capacity, poor thermal conductivity, etc., through surface modification or compounding of multiple NPs for the purpose of improving the characteristic properties of nanoparticles.

**Figure 10:**
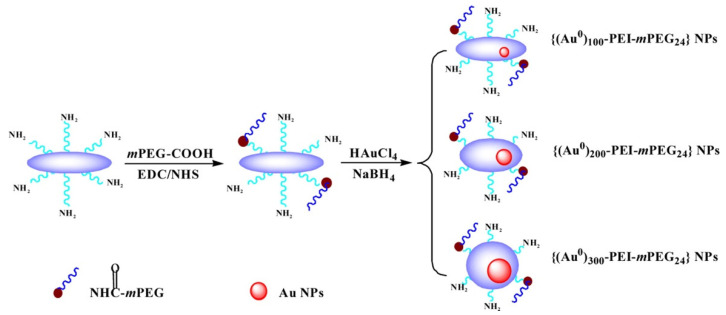
Schematic illustration of the synthesis of PEG-Au PENPs. (Reproduced with permission from Ref. [55], Copyright 2016, American Chemical Society).

Although Au NPs have been used maturely in PCR, it has been found that surface-modified Au NPs have also had a strong enhancement effect on PCR in recent years. In addition, some Au modified complexes have further specific effects on PCR. Chen et al. synthesized the dendrimer-entrapped Au NPs (Au DENPs) using amine-terminated G5 dendrimers as templates and different compositions as additives to investigate their effects on the specificity and efficiency of PCR amplification. It was found that the optimum concentration of Au DENPs could be reduced to as low as 0.37 nM, much lower than that of NH_2_-G5 dendrimers without Au NPs entrapped [54]. One year later, using poly (diallyl dimethylammonium) chloride (PDDA) as novel PCR enhancers, Yuan et al. verified the improvement of three kinds of Au NPs modified with different surface charge polarities in the efficiency and specificity of an error-prone two-round PCR system. The optimum concentrations of positively charged PDDA-Au NPs were different and as low as 1.54 pM, while the negatively charged Na_3_Ct-Au NPs were over three orders of magnitude higher than the positive ones [11]. Additionally, polyethylene glycol (PEG)-modified polyethylenimine (PEI)-entrapped Au NPs (PEG-Au PENPs) showed potential capacity to enhance the specificity and efficiency in both two-round PCR and GC-rich PCR. As the proportion of gold content increased, the optimum concentration of the modified Au NPs decreased (Figure 10) [55].

**Figure 11:**
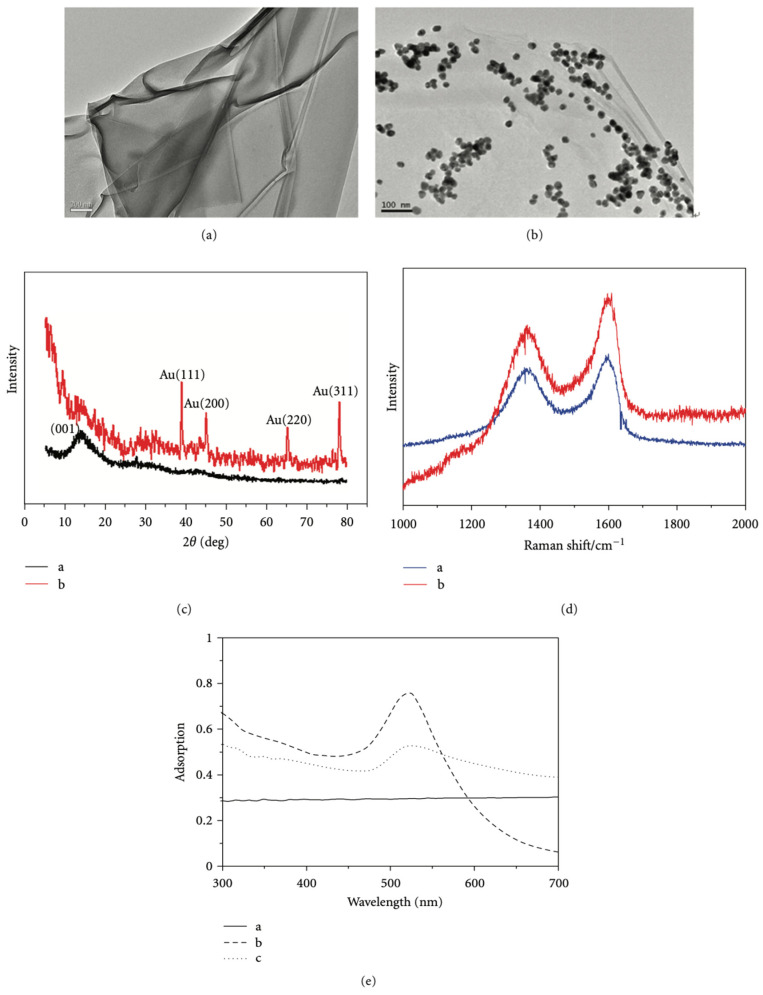
(**a**,**b**) TEM images of the GO and GO-Au composites, respectively. (**c**) XRD images of the GO and GO-Au composites. (**d**) Raman spectra of the GO and GO-Au composites. (**e**) UV-vis absorption spectra of the GO, Au NPs and GO-Au composites from a to c. (Reproduced with permission from Ref. [57], Copyright 2012, Hindawi).

Some nanomaterials can be effectively applied to PCR, but there are always some limitations. For example, despite the merits and capabilities of GO, a severe agglomeration level leads to a limited surface area, which may impede PCR performance. The modification of GO with Au NPs overcomes these challenges as hybrid nanomaterials maintain the beneficial features of both precursor materials and provide advantages unique to the hybrid material through the combination of functional components (Figure 11). Jeong et al. [56] synthesized an Au NP and GO hybrid composite and applied it as a PCR enhancer.

Dao Van et al. [58] successfully synthesized Fe_3_O_4_/SiO_2_ NPs consisting of a 10–15 nm core and a 2–5 nm thick silica shell. The Fe_3_O_4_/SiO_2_ NPs were found to be more efficient at purifying DNA from HBV and EBV than using commercial Fe_3_O_4_/SiO_2_ particles, as indicated by (i) brighter PCR amplification bands for HBV and EBV viruses and (ii) higher sensitivity for PCR-based EBV loading detection.

#### 2.5.2. Other NPs

Metal-organic frameworks (MOFs) are special organic–inorganic hybrid porous solids with extraordinarily high surface areas, tunable pore sizes, adjustable internal surface properties, and an extraordinary degree of variable structures. These features endow MOFs with potential gas or liquid adsorption/storage applications, such as drug delivery, polymerization, catalysis, and biosensors. Recently, Sun et al. used UiO-66 and ZIF-8 to optimize the error-prone two-round PCR and found that both UiO-66 and ZIF-8 not only enhanced the sensitivity and efficiency of the first-round PCR but also increased the specificity and efficiency of the second-round PCR. Moreover, both MOFs could widen the annealing temperature range of the second-round PCR [59]. Also, Rasheed et al. developed a hexagonal boron nitride (hBN) NP-based PCR assay for the rapid detection of Acanthamoeba to amplify DNA from low amoeba cell density. As low as 1 × 10^−4^ (wt%) was determined as the optimum concentration of hBN NPs, which increased Acanthamoeba DNA yield up to ~16%. Further, it was able to reduce PCR temperature, which led to a ~2.0-fold increase in Acanthamoeba DNA yield at an improved PCR specificity at 46.2 °C low annealing temperature. hBN nanoparticles further reduced standard PCR step time by 10 min and cycles by 8 min, thus enhancing Acanthamoeba detection rapidly [60].

## 3. The Effects of NPs in Real-Time PCR

Real-time PCR is routinely used in molecular biology labs just like conventional PCR. Its advantages over conventional PCR include the ability to visualize reactions that have worked in real time and without the need of an agarose gel. It also allows truly quantitative analysis. One of the most common uses of real-time PCR is to determine the copy number of a DNA sequence of interest. Using absolute quantitation, the user is able to determine the target copy numbers in reference to a standard curve of defined concentration in a far more accurate way than ever before. Here we discuss the effect of some NPs across the real-time PCR amplification process [61].

Namadi et al. [62] did a very meaningful study in which they used gold NPs to bind to targets to show overexpression of follistatin-related protein 1 (FSTL1) and FSTL3 in heart failure (*p* < 0.05) by real-time PCR. The data showed that the elevated expression of FSTL1 and FSTL3 was a marker of heart failure and was detected within 30 min by synthetic Au NPs, which was accurate and rapid.

**Figure 12:**
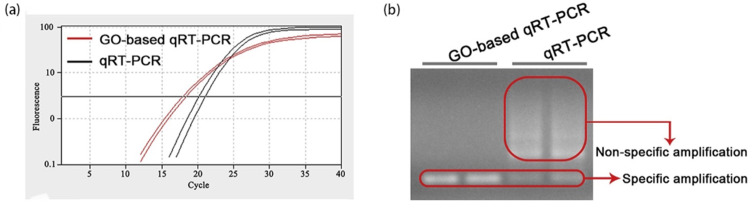
(**a**) Amplification curves and (**b**) Agarose gel electrophoresis images of GO-based qRT-PCR and conventional qRT-PCR assays. (Reproduced with permission from Ref. [63], Copyright 2021, Elsevier).

Hu et al. [63] also investigated GO-based qRT-PCR detection methods, which confirmed that GO could reduce the occurrence of non-specific amplification by non-covalent interaction with primers and ssDNA, significantly improving the sensitivity and specificity of qRT-PCR detection. As shown in Figure 12, compared with conventional qRT-PCR, the Ct value of the GO-based qRT-PCR significantly decreased (*p* < 0.05) (Figure 12a). Furthermore, the results of agarose gel electrophoresis confirmed that the GO-based qRT-PCR exhibited no non-specific amplification, while the conventional qRTPCR displayed apparent non-specific band amplification (Figure 12b).

At present, one of the most popular techniques in PCR and real-time PCR detection is the separation and purification of DNA by magnetic NPs to improve detection sensitivity [64,65]. For example, Yang et al. developed a method combining nanoparticle-based immunomagnetic separation (IMS) and real-time PCR for the rapid and quantitative detection of Listeria monocytogenes. Carboxyl modified magnetic NPs were covalently bound with rabbit anti-*L. monocytogenes* via the amine groups. *L. monocytogenes* DNA ≥ 10^2^ CFU/0.5 mL was detected in milk samples containing *L. monocytogenes*, ranging from 10^3^ to 10^7^ L. The number of cells calculated based on the C_T_ value is 1.5 to 7 times that of the plate count. The results showed that both the use of NPs and the choice of anti-*L. monocytogenes* in the IMNP-based IMS in combination with real-time PCR has improved the sensitivity of *L. monocytogenes* detection from both nutrient broth and milk samples [66]. Bakthavathsalam et al. [67] developed a rapid and sensitive method for immunomagnetic separation (IMS) of *Salmonella* along with their real time detection via PCR. Silica-coated magnetic NPs were functionalized with carboxy groups to which anti-*Salmonella* antibodies raised against heat-inactivated whole cells of *Salmonella* were covalently attached. The immune-captured target cells were detected in beverages like milk and lemon juice by multiplex PCR and real-time PCR with a detection limit of 10^4^ cfu·mL^−1^ and 10^3^ cfu·mL^−1^, respectively. Zhong et al. [68] extracted DNA by magnetic particles to produce high-quality DNA for real-time quantitative PCR using an optimized set of primers. The method was highly sensitive, as it was capable of detecting as little as 100 cfu of *P. aeruginosa*. It was also highly specific, as DNA extracted from other species of bacteria did not generate positive signals. Yuan et al. similarly used magnetic beads to isolate DNA from affected periodontal tissue and detect *Porphyromonas gingivalis* DNA by routine or quantitative real-time PCR, which has been shown to be specific, sensitive, and accurate [69]. Ernst et al. optimized the method for extracting and purifying methicillin-resistant *Staphylococcus aureus* (MRSA) DNA with magnetic NPs, and it could save approximately 20 min [70]. Wu et al. [71] used protamine-coated magnetic NPs (PMNPs) to capture suspended viral particles, a process that led to a selective concentration of viral particles, which could subsequently be quantified for real-time PCR analysis. A sensitive real-time PCR detection method was established.

**Figure 13:**
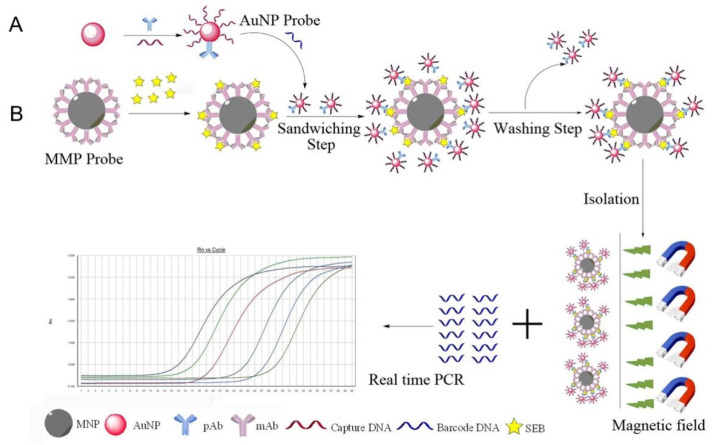
Schematic of SEB analysis based on the BCA. (**A**) Preparation of pAb–AuNP–DNA barcode probes. (**B**) Principles of BCA combined with real-time PCR. (Reproduced with permission from Ref. [72], Copyright 2019, Elsevier).

Xu et al. [72] developed an ultrasensitive method involving (1) Au NPs encoded with double-stranded DNA as the first signal amplification and goat anti-staphylococcal enterotoxin B (SEB) polyclonal antibodies and (2) magnetic microparticles coated with anti-SEB monoclonal antibodies to detect SEB. Both functionalized nanoparticles can capture SEB in a sandwich system. The released DNA barcodes were then characterized through SYBR Green real-time PCR and resulted in the second signal amplification (Figure 13). Its detection limit could reach 0.269 pg mL^−1^, which was 1000-fold lower than that of conventional enzyme-linked/immunosorbent assay.

## 4. Mechanisms of Nanomaterials in PCR

Table 1 summarizes the enhancement effects and mechanisms of different nanomaterials one by one.

### 4.1. Surface Interactions

**Figure 14:**
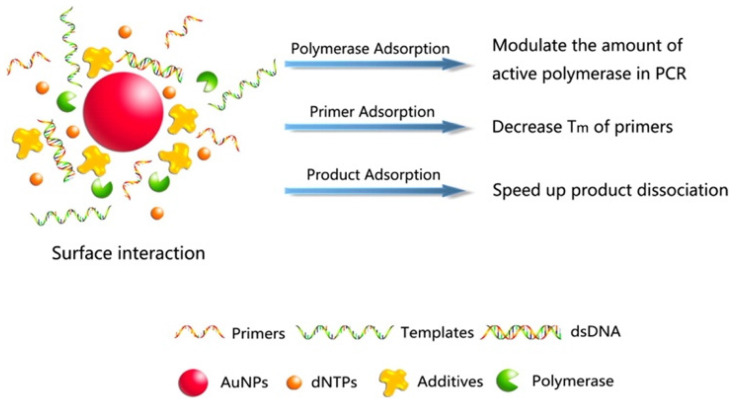
Schematic illustration of the surface interactions between Au NPs and PCR components (Reproduced with permission from Ref. [22], Copyright 2013, American Chemical Society).

For metal-nanomaterials, a possible mechanism was proposed that Au NPs might modulate the activity of polymerase to improve PCR amplification [76], effectively reducing polymerase concentration to suppress the amplification of longer products while favoring amplification of shorter products through nonspecifically adsorbing polymerase in the nanoparticle absorption spectrum and electrophoretic mobility in the presence of a polymerase [19]. Lou et al. [22] reported that nanoPCR could be regulated by the surface interactions between not only NPs and polymerases but also primers and products absorbed by metal-nanomaterials, as shown in Figure 14. Similar to Au NPs, the adsorption of polymerase, primers, and templates by nano-silver was claimed to be the main reason for the inhibition of DNA synthesis [25].

Cui et al. [7] found that the DNA templates and Taq enzymes were attached to the bundles of SWCNTs in PCR products for carbon-based nanomaterials. The possible mechanism could be the aggregation of reaction components caused by the van der Waals attraction. MWCNTs with DNA and enzymes were pointed out to prevent their further agglomeration through strong physical interactions [30]. Zhang et al. indicated that carbon-based nanoparticles could directly bind with DNA molecules to improve the PCR efficiency observed by atomic force microscopy [33]. The interaction between rGO and Pfu DNA polymerase was proved to play a dominant role in improving the specificity of PCR [36]. In addition, Wang et al. monitored the interactions between GO and PCR components using a capillary electrophoresis/laser-induced fluorescence polarization (CE-LIFP) assay and found that the addition of GO promoted the formation of a matched primer–template complex but suppressed the formation of a mismatched primer–template complex during PCR, which revealed the essential role the interactions between the primers and GO played in enhancing PCR specificity [8].

**Figure 15:**
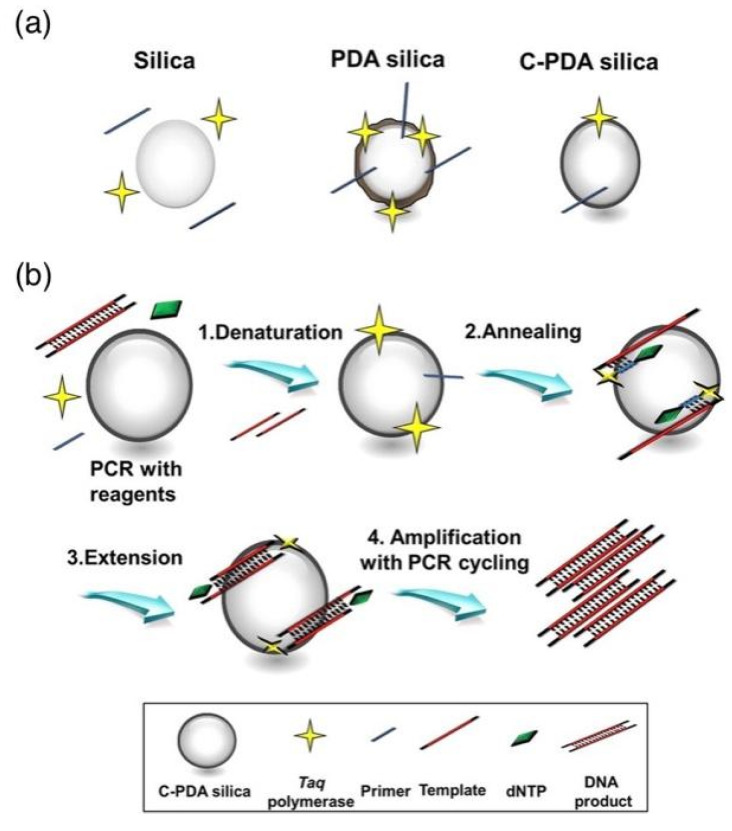
(**a**) C-PDA silica provided binding sites to immobilize the primers and polymerase and (**b**) C-PDA silica in the PCR process. (Reproduced with permission from Ref. [49], Copyright 2015, American Chemical Society).

As for oxide nanomaterials, the primary reasons allowing Fe_3_O_4_ NPs to outperform Au and Ag NPs seemed to be attributed to the effective adsorption of PCR components onto the ammonium salt of oleic acid-coated magnetite nanofluids [45]. The effects of C-PDA silica on PCR were observed by employing as-prepared silica and PDA silica so as to investigate the interaction between the materials and PCR reagents. The substantial negative charges of silica showed almost no interaction with primers nor polymerase. By contrast, the PDA silica provided numerous binding sites to immobilize the primers and polymerase on the surface to enhance the stability. Moreover, C-PDA silica allowed the mild interaction with primers and polymerase but addressed the best PCR enhancement (Figure 15) [49].

The effect of the QDs was optimized to be the affinity between the DNA polymerase and the QDs, as the DNA polymerase could be adsorbed onto the QDs, causing a reduction in the effective concentration of the polymerase in the PCR system. Therefore, only the target PCR product, most efficiently annealed with primers, would be amplified preferentially under these conditions. More QDs were added, and more polymerases were adsorbed. With adequate QDs added to the PCR system, the polymerase concentration decreased and was less than the optimal effective concentration for specific amplification [6]. The study on the interactions of the primers and Mg^2+^ with GQDs in PCR found that the primer stacking on GQDs improved the sensitivity and specificity of PCR by improving the efficiency of base-pairing between the primers and the templates. The PCR yield was improved primarily by GQDs via increasing polymerase activity, where GQDs were tuned through chelating Mg^2+^ with their peripheral carboxylic groups [52].

**Figure 16:**
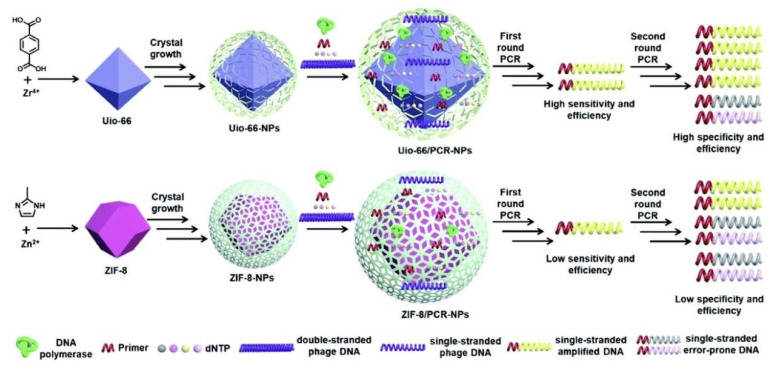
The possible interaction among templates, Taq polymerase and UiO-66 (or ZIF-8) during PCR. (Reproduced with permission from Ref. [59], Copyright 2019, Royal Society of Chemistry).

In addition, Au/GO hybrid composites were synthesized and used in PCR. It was proven that the interaction among ssDNA, primer, polymerase, and graphene-based materials was mainly attributed to π-π stacking and electrostatic attraction, which improved the stability of the PCR components, including DNA, polymerase, and primer, making the Au/GO as an ideal PCR enhancer [56]. Recently, Sun et al. introduced MOFs like UiO-66 and ZIF-8 into PCR and proposed that the main reason for MOFs increasing the specificity and efficiency in two-round error PCR might be the interaction of DNA and Taq polymerase with MOFs (Figure 16) [59].

### 4.2. Thermal Conductivity

Yan et al. demonstrated that the thermal conductivity enhanced by Au NPs was the primary mechanism for the increasing PCR efficiency and specificity [21]. Jia et al. also pointed out that rGO had an unusually high thermal conductivity (5300 WmK^−1^ ) and suggested that the rGO-assisted PCR system could rapidly reach thermal equilibrium during the heating/cooling processes [36]. Like graphene, GNFs with enlarged surface area increased the heat conductivity to produce high thermal conductivity for the final purpose of enhancing PCR [37].

On the other hand, Abdul et al. [41] investigated the mechanism of PCR enhancement by simulations using the Fluent K epsilon turbulent model, providing evidence of faster heat transfer in the presence of TiO_2_ NPs [41]. In 2016, Kambli et al. compared the enhanced PCR efficiency from three transition metal NPs in the form of stable colloidal suspensions at varying concentrations and found that the enhancing rate of the ammonium salt of oleic acid-coated magnetite NPs scored highly over that of Au and Ag NPs at a 10^−2^ times less concentration owing to their cluster and particle alignment properties that enhanced thermal conductance, though magnetite had the least thermal conductivity [45].

### 4.3. Electrostatic Interactions

Usually, there is an electrostatic interaction between the positive and negative charges on their surface as the gold is modified on the polymer macromolecules. PCR components played an essential role in improving the specificity and efficiency of PCR [11,55,56].

Table 2 shows that PEG−Au PENPs are exposed to more terminal amino groups on the surface as the gold loading content increases, providing more opportunities for reactions between NPs and PCR components, resulting in a decrease in the optimal concentration used in error-prone two-round PCR systems. However, due to the decrease in the number of amine terminal groups, the PCR enhancement effect is weakened after surface acetylation of PEG−Au PENPs. It can be inferred that the electrostatic interaction between positively charged NPs and negatively charged PCR components has a great influence on improving the specificity and efficiency of PCR.

As reported, the required cycling time of the PCR was shortened dramatically owing to the addition of MWCNTs, CNT/PEI, and PEI, suggesting that PCR improvement should not solely depend on the rapid heat exchange in the presence of CNTs. Notably, the interaction between the PCR components and the positively charged PEI or CNT/PEI should play a crucial role in improving PCR specificity and efficiency [29]. Moreover, three types of CNTs synthesized with different surface charges displayed different effects on enhancing PCR. It was found that only CNTs functionalized with pristine and amine groups could enhance PCR, while the carboxylated CNTs inhibited PCR in all samples, which might be caused by the electrostatic repulsion between negative charges [31]. Jia et al. demonstrated that the ultimate positively charged complex of rGO-Pfu would be beneficial to attract the negative charged DNA templates and primers onto the rGO plate and promote primer annealing and extension [36].

The conjugation of ZnO tetrapods with plasmid DNA was evaluated by agarose gel electrophoresis based on the electrostatic interactions between the positively charged amino groups on tetrapods and the negatively charged phosphate groups of plasmid DNA. Unlike the covalent bonding, these electrostatic interactions were weak, and the conjugation of ZnO tetrapods with DNA was reversible. The tetrapods could thus be used for the purification of plasmid DNA in cell lysates [10].

### 4.4. Analogs to ssDNA Binding Protein (SSB)

Nanomaterials mimic the function of SSBs to selectively bind single-stranded DNA (ssDNA) rather than double-stranded DNA (dsDNA). For example, Wang et al. [6] attributed the optimization effect of the QDs on the specificity of the PCR to the similar optimization mechanism of the ssDNA-binding SSB, which selectively bound to the ssDNA rather than dsDNA and then minimized the mispairing between the primers and the templates in the PCR system. Two reasons were summarized as follows: First, the surface of the QDs used in this study was modified, with the carboxyl groups responsible for the negatively charged surface of the QDs. The dsDNA with a higher surface charge density was more repulsive than the ssDNA in the negative atmosphere. Thus, more negatively charged QDs bond quickly to the anionic ssDNA strands rather than to the dsDNA, similar to the way that SSB protein selectively binds to the ssDNA. Second, the dsDNA rigidity did not favor the wrapping of the dsDNA around the QDs, while the ssDNA was a soft and flexible polymer with a much greater degree of freedom to wrap around the QDs. Such selectivity greatly minimized the mispairing between the primers and the templates during DNA replication, similar to the SSB.

### 4.5. Catalytic Activity

Catalytic activity refers to the ability of nanomaterials to enable PCR to proceed even when the environmental conditions are not the best fit.

The CNTs are well known to possess catalytic properties. Cui et al. [7] investigated the effects of SWCNTs on PCR via the quantitative PCR product measurements and some other techniques. Similar results in PCR reactions were obtained in the presence and the absence of Mg^2+^ serving as electron donors/receptors. Au NPs were verified to exhibit ‘mimic enzyme’ catalytic activity under certain conditions as well [22].

## 5. Application and Prospect of NanoPCR

NanoPCR has the advantages of high sensitivity, specificity, and selectivity, and has been widely used in bacteria, virus, tumor detection, vand new detection platforms. Table 3 shows the application of nanoPCR in different fields in the past decade.

Gabriel et al. [75] developed a nanoPCR assay for the rapid detection of brain-eating amoeba using GO, CuO, and Al_2_O_3_ NPs. The results showed that the three NPs significantly improved the PCR efficiency of detecting pathogenic free-living amoeba using genus-specific probes. Moreover, the combinations of these NPs proved to further enhance PCR efficiency. The addition of metal oxide NPs leads to excellent surface effect, while thermal conductivity property of the NPs enhances PCR productivity. These findings suggest that nanoPCR assay has tremendous potential in the clinical diagnosis of parasitic infections as well as for studying epidemiology and pathology and environmental monitoring of other microbes.

At present, nanoPCR has been widely used in diagnosing animal diseases and detecting various viruses. For instance, Wang et al. [78] detected porcine bocavirus (PBoV) based on the nanoPCR. The assay was 100-fold more sensitive than the conventional PCR assay, with the detection limit of about 6.70 × 10^1^ copies. Yuan et al. [79] used the nanoPCR technique to detect the porcine epidemic diarrhea virus (PEDV) for the first time, obtaining a 100-fold more sensitive assay than conventional RT-PCR. The limit of detection was 2.7 × 10^−6^ ng/μL of PEDV RNA with no cross-reaction observed in the presence of other viruses.

**Figure 17:**
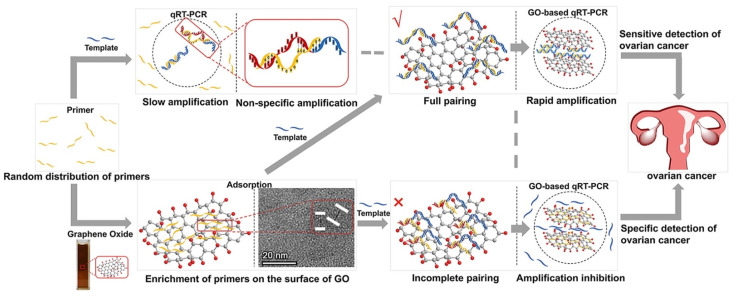
The process for the detection of miRNAs associated with OC in conventional qRT-PCR and GO-based qRT-PCR. (Reproduced with permission from Ref. [63], Copyright 2021, Elsevier).

For the early diagnosis and therapy of cancer, Hu et al. designed and developed a GO-based qRT-PCR assay for the detection of miRNAs associated with ovarian cancer (OC) (Figure 17). The detection of miRNAs associated with OC confirmed that the GO-based qRT-PCR assay could differentiate benign ovarian tumors from OC (sensitivity, 0.91; specificity, 1.00).

In recent years, with the outbreak of the COVID-19 epidemic, many researchers have begun to study the quick and convenient methods of nucleic acid testing to control the spread of the virus as quickly as possible. For example, Lee et al. [95] developed two new points of care (POC) tests to enable the rapid diagnosis of infection. One of them is the nanoPCR that takes advantages of core−shell magnetoplasmonic nanoparticles (MPNs): (i) the Au shell significantly accelerates thermocycling via volumetric, plasmonic, light-to-heat conversion, and (ii) a magnetic core enables sensitive in situ fluorescent detection via magnetic clearing. When applied to COVID-19 diagnosis, nanoPCR detected SARS-CoV-2 RNA down to 3.2 copy/μL within 17 min. In particular, nanoPCR diagnostics accurately identified COVID-19 cases in clinical samples (n = 150), validating its clinical applicability.

In short, nanoPCR technology has opened up a new way to study biomolecules with crucial applications in practical research, especially in virus detection and new PCR detection platform. In the future, nanoPCR will have good application prospects in the field of biomedicine. However, due to the complexity of the PCR reaction system and the characteristics of NPs, the mechanism of nanoPCR is still unclear and needs more exploration. Therefore, it is of great significance to study the reaction mechanism of nanoPCR, and the development of non-toxic and efficient nanomaterials is a significant direction for future research.

## 6. Conclusions

Because of the unique physical and chemical properties, nanomaterials have been steadily and reasonably used in PCR to improve efficiency and specificity. Compared with traditional PCR technology, The nanomaterials with excellent surface properties, thermal conductivity, and catalytic activity introduced into the PCR system can effectively shorten the reaction time, increase the amplification efficiency and specificity, increase the product yield, widen the annealing temperature range, and greatly improve the detection sensitivity. According to the DNA templates, the nanomaterials modified with primers, polymerases, and Mg^2+^ on the surface can improve the reaction efficiency significantly. With the continuous development of nanomaterials and PCR, the mechanism study is becoming more and more precise, but further in-depth research is still needed to make the mechanism clearer. In addition, the impact on PCR efficiency is often the joint result of the simultaneous functioning of many different mechanisms, requiring full consideration of all possible factors. Therefore, the nanoPCR technology has opened up a new way to study biomolecules.

## Figures and Tables

**Table 1:** Reports of nanomaterials enhancing polymerase chain reaction.

Category	Material	Type of DNA Molecule	Mechanism	Effect	References
Metal nanomaterials	Au NPs	cDNA from bladder cancer cell line and lung cancer tissue, BNIP3 cDNA from bladder cancer cells and colorectal tissue	Thermal conductivity	Increase the yield by 10^4^–10^6^ times; Shorten the reaction time	[17]
		309 bp fragment from pBR322 DNA	Surface interactions	Increase the yield of PCR product; Specificity; Efficiency	[18]
		283-bp λ-DNA	/	Enhance sensitivity and specificity in multi-round PCR	[19]
		309 bp λ-DNA and genomic cDNA	Surface interactions	Specificity; Efficiency (favor smaller products)	[20]
		pBR322 DNA template	Thermal conductivity	Specificity; Efficiency	[21]
		Human male genomic DNA	Surface interactions; Catalytic activity	Specificity; Efficiency	[22]
		Housekeeping gene GAPDH from the human DNA template	/	Increase the yield of PCR product	[23]
	Au DENPs	283-bp λ-DNA	Electrostatic interactions	Specificity; Efficiency	[54]
	PDDA-Au NPs	283-bp λ-DNA	Electrostatic interactions	Specificity; Efficiency	[11]
	PEG−Au PENPs	283-bp λ-DNA	Electrostatic interactions; Thermal conductivity	Specificity; Efficiency	[55]
	Ag NPs	g-DNA, λ-DNA (kb)	Thermal conductivity	Increased PCR efficiency with long DNA and repeated amplification	[24]
		714 bp GFP gene	Surface interactions	Inhibition	[25]
		750 bp mCherry containing plasmid	/	Efficiency	[16]
Carbon-based nanomaterials	CNTs	410 bp DNA	Surface interactions; Catalytic activity	Increase the yield of PCR product	[7]
		14.3 kb λ-DNA	/	Specificity; Efficiency	[28]
	CNT/PEI	283 bp λ-DNA	Electrostatic interactions; Thermal conductivity	Specificity; Efficiency	[29]
	NH_2_-MWCNTs	94 mer random DNA oligonucleotide library	Surface interactions	Specificity; Efficiency (filtered NH_2_-MWCNT)	[30]
	SWCNTs, NH_2_-SWCNTs	283 bp λ-DNA	Electrostatic interactions	Increase the yield of PCR product	[31]
	CNP	540 bp g-DNA	Surface interactions	Increased PCR specificity and efficiency with long DNA and repeated amplification	[33]
	Graphene	300 bp fragment from pET-32a plasmid DNA	Surface interactions; Electrostatic interactions; Thermal conductivity	Specificity	[36]
		283 bp λ-DNA	Surface interactions	Specificity	[8]
	GNFs	1248 bp g-DNA	Thermal conductivity	Reduce cycles, Efficiency	[37]
	GO	pET-32a plasmid	Electrostatic interactions	Specificity	[73]
Oxide nanomaterials	TiO_2_	650 bp DNA	Surface interactions	Inhibition	[9]
		Mouse and human genomic DNA, plasmid DNA, and mouse complementary DNA [cDNA]	Thermal conductivity	Efficiency	[41]
		cDNA or gDNA	/	Increase the yield of PCR product	[42]
	Silica-coated and amino-modified ZnO	Plasmid DNA	Electrostatic interactions	Increase the yield of PCR product	[10]
	ZnO	619 bp and 666 bp DNA	/	Specificity; Efficiency; Reduce reaction time	[74]
	Fe_3_O_4_	800 bp prokaryotic DNA	Surface interactions; Thermal conductivity	Efficiency	[75]
	MgO	/	/	Efficiency	[48]
	SiO_2_	Genomic DNA of *E. coli* (eae1, 248 bp) and pEGFP-C1 plasmid (egfp, 800 bp)	Surface interactions	Increase the final quantity of PCR product	[49]
Fluorescent nanomaterials	CdTe QDs	λ-DNA	Analogous to ssDNA binding protein (SSB); Surface interactions	Specificity	[6]
		1000 bp human genomic DNA	Surface interactions	Reduce reaction time	[50]
		Human DNA, plasmid DNA or marine fouling organism DNA	Surface interactions	Retained specificity in the ninth-round amplification	[51]
	GQDs	80 bp fragment from a GC-rich DNA	Surface interactions	Specificity; Efficiency; Increase the yield of PCR product	[52]
	UCNPs	120 bp 5S rRNA	/	Specificity	[53]
Others	GO-Au composites	Genomic DNA of Listeria monocyte (200 bp) and Scomber japonicas (800 bp)	Surface interactions	Specificity; Efficiency; Broad annealing temperatures	[56]
	MOFs	λ-DNA	Surface interactions	Specificity; Efficiency; Wide annealing temperatures	[59]

**Table 2:** Physicochemical properties and optimum concentrations of the additives in the error-prone two-round PCR. (Reproduced with permission from Ref. [55]., Copyright 2016, American Chemical Society).

Additives	ζ-potential (mV)	Optinimum Concentration (mg/L)	Maxima Efficiency ^a^	Maximal Specificity ^a^
PEI	24.07 ± 1.45	0.47	1.5	1
{(Au^0^)_100_-PEI-*m*PEG_24_} NPs	28.93 ± 0.85	0.38	2.2	1
{(Au^0^)_200_-PEI-*m*PEG_24_} NPs	33.46 ± 1.28	0.34	3.6	1
{(Au^0^)_300_-PEI-*m*PEG_24_} NPs	34.23 ± 1.09	0.38	1.9	1
{(Au^0^)_200_-PEI·NHAc-*m*PEG_24_} NPs	6.34 ± 1.13	60	1.4	1

^a^ Depends on the performance of each additive with optimum concentration.

**Table 3:** The applications of nanoPCR in different fields.

Category	Type or Purpose of Detection	NPs	Effect	References
Bacteria detection	Strain Typing of *Salmonella typhi*	Citrate stabilized Au NPs, rhamnolipid stabilized Au and Ag NPs, and magnetic iron oxide NPs	Reduce non-specific amplification (Au and Ag NPs); Increase PCR yield (Au NPs, Au and Ag NPs); Inhibition (magnetic iron oxide NPs)	[73]
	Bacterial aerosols	Ag NPs, TiO_2_ NPs and their combination	The detection limit down to 40 pg/μL	[74]
	Brain-eating amoebae	GO, CuO and Al_2_O_3_ NPs	Enhanced PCR efficiency	[75]
Virus detection	Porcine parvovirus	Solid NPs (1–100 nm diameter)	Enhanced PCR sensitivity (100-fold more sensitive)	[76]
	Detection and differentiation of wild-type pseudorabies virus and gene-deleted vaccine strains	Solid Au NPs (1–100 nm)	Enhanced PCR sensitivity (100–1000-fold more sensitive)	[77]
	Porcine bocavirus	Solid Au NPs (1–100 nm) form colloidal nanofluids	Enhanced PCR sensitivity (100-fold more sensitive); The detection limit down to 6.70 × 10^1^ copies	[78]
	Porcine epidemic diarrhea virus	Solid Au NPs(1–100 nm) form colloidal nanofluids	Enhanced PCR sensitivity (100-fold more sensitive); The detection limit down to 2.7 × 10^−6^ ng/μL	[79]
	Mink enteritis virus (MEV)	No instructions	The detection limit down to 8.75 × 10^1^ copies recombinant plasmids per reaction	[80]
	Concurrent infections of pseudorabies virus and porcine bocavirus	Solid Au NPs (1–100 nm) form colloidal nanofluids	Enhanced PCR efficiency; The detection limit of 6 copies for PRV and 95 copies for PBoV	[81]
	A diagnostic technique for equine herpes virus-1 (EHV-1)	Au NPs	Increase PCR yield; The detection limit down to 10^2^ DNA copies	[82]
	Encephalomyocarditis virus	Solid Au NPs(1–100 nm) form colloidal nanofluids	Enhanced PCR sensitivity and specificity;Detection limit down to 1.2 × 10^2^ copies/μL	[83]
	Porcine epidemic diarrhea virus and porcine transmissible gastroenteritis virus	Solid NPs (1–100 nm diameter)	Enhanced PCR sensitivity (10-fold more sensitive)	[84]
	Bovine respiratory syncytial virus	Au NPs	Enhanced PCR sensitivity; Detection limit down to 1.43 × 10^2^ copies recombinant plasmids per reaction	[85]
	Bovine Rotavirus, Bovine Parvovirus, and Bovine Viral Diarrhea Virus	Au NPs	Enhanced PCR sensitivity and specificity	[86]
	Quick Diagnosis of Canine Vector-Borne Pathogens	ZnO Nanoflower	Reduce the reaction time; Enhanced PCR sensitivity and specificity	[44]
	HPV-16 and HPV-18 DNA	Solid Au NPs(1–100 nm)	Enhanced PCR sensitivity (10-fold more sensitive) and specificity	[87]
	Distinguishing canine coronaviruses I and II	Solid Au NPs (1–100 nm) form colloidal nanofluids	Enhanced PCR sensitivity (100-fold more sensitive) and specificity	[88]
	Canine distemper virus (CDV), canine parvovirus (CPV) and canine coronavirus (CCV)	Solid Au NPs(1–100 nm)	Enhanced PCR sensitivity and specificity	[89]
	Goose Parvovirus	Au NPs	Enhanced PCR sensitivity (100-fold more sensitive)	[90]
	Feline calicivirus, feline panleukopenia syndrome virus, and feline herpesvirus type I virus	Au NPs	Enhanced PCR sensitivity (10–100-fold more sensitive) and specificity	[91]
Tumor monitoring	Single-base mutations to monitor tumor	Au NPs	Enhanced PCR sensitivity and specificity	[92]
	Detection of miRNAs to screen ovarian cancer	GO	Enhanced PCR sensitivity and specificity	[63]
No machine PCR	Plasmonic photothermal gold bipyramid banoreactors	Gold bipyramid nanoparticles (Au BPs)	Achieved ultrafast thermocycling	[93]
	To realize on-site and instant analysis	GO, rGO, molybdenum disulfide (MoS_2_), and tungsten disulfide (WS_2_)	Achieved visual detection (MoS_2_ and WS_2_)	[94]
	point of care (POC) settings	Core−shell magnetoplasmonic nanoparticles (MPNs)	Detected SARS-CoV-2 RNA down to 3.2 copy/μL within 17 min	[95]
	Detection of health-related DNA and proteins	Au NPs	High sensitivity, visual detection, capability for on-site detection	[14]
	Real time label-free monitoring of plasmonic	Au NPs	The detection limit down to 10,000 genome copies/μL	[96]
	Diagnosis of Hepatitis C Virus	Streptavidin-coated magnetic particles (1μm) and anti-digoxigenin antibody-coated polystyrene particles (250–350 nm)	Visual detection; High sensitivity and specificity	[97]

## Data Availability

This is a review paper, and no data included.
